# Gut‐Microbiome and Nutritional Analysis Reveals Food Intake as a Key Factor in the Incidence and Prevention of Colon Polyps: A Cross‐Sectional Study

**DOI:** 10.1002/hsr2.71109

**Published:** 2025-08-26

**Authors:** Chuanmin Ma, Mingbao Zhang, Binbin Chen, Ming Liu, Huiyu Jiang, Jiaorong Li, Xintong Song, Xiangrui Wei, Zhixing Wei, Jingyao Liu, Hengyun Guan, Jun Zhou, Hui Liu

**Affiliations:** ^1^ Jinan Municipal Center for Disease Control and Prevention Affiliated to Shandong University Jinan China; ^2^ Department of Gastroenterology, the Second Hospital of Shandong University Shandong University Jinan China; ^3^ Cheeloo College of Medicine Shandong University Jinan China; ^4^ Department of Toxicology, Shandong Academy of Occupational Health and Occupational Medicine Shandong First Medical University Jinan China

**Keywords:** colon polyps, dietary analysis, disease etiology, gut‐liver axis, gut‐microbiome

## Abstract

**Background and Aims:**

Dietary patterns and associated gut microbiota are increasingly recognized as key contributors to the development and prevention of colon polyps (CP). This study aimed to investigate whether specific dietary components and gut microbiome alterations are associated with CP incidence and severity, and to compare these patterns with other gastrointestinal or hepatic conditions.

**Methods:**

Stool samples were collected from individuals with CP, healthy controls, and patients with alcoholic fatty liver disease (AFLD), metabolic‐associated fatty liver disease (MAFLD), and ulcerative colitis (UC). Gut microbiome profiling was conducted to characterize microbial composition across groups. Dietary intake data were analyzed, with a focus on nutrient profiles and potential food contaminants. CP patients were further divided into subgroups based on polyp number (single vs. multiple) for dietary pattern comparison.

**Results:**

Distinct microbiota profiles were observed among groups: Bacteroidetes dominated in CP patients, Actinobacteria in AFLD, Proteobacteria in MAFLD, and Firmicutes in UC. CP‐associated microbiota were enriched in Prevotellaceae and Paraprevotellaceae. Dietary patterns linked to CP included high‐fat, ketogenic, and high‐sugar diets, as well as possible exposure to food contaminants. Patients with multiple polyps exhibited higher intake of calories, fat, and red meat, while those with single polyps consumed diets lower in calories and fat but richer in vitamins E and K.

**Conclusion:**

Food intake is strongly associated with both the incidence and severity of colon polyps, likely through modulation of the gut microbiome and nutritional environment. These findings support the potential for dietary interventions targeting gut microbial composition to prevent or mitigate CP development.

## 1 Introduction

Colon polyp is mushroom‐like proliferative tissue bulging from the lining of colon or rectum as a result of uncontrollable proliferation of epithelial cells. Currently, colon polyps (CP) disease is of highly prevalence in American adults, reaching an estimated prevalence of 15% and 40% of adults [1]. In China, CP prevalence is estimated to be 18.1% in the adults aged from 47–67, based on a surveillance of 3066 subjects during 2009–2013 [2]. Though colon polyp can be removed with colposcopy, it frequently recurs in later life time of patients. According to statistics, the cumulative recurrence rate of colon polyp was 13.8% for 1 year after medical removal and 60% for 3 years after medical removal [3]. In the Western nations, a number of studies have investigated the etiology and risk factors of CP [4, 5, 6]. The particular mechanistic cause of CP is not fully understood but available evidence suggest that it is associated with family history (genetics) [7], aging [8], obesity [9], smoking, and drinking alcohol [10, 11]. It is also suggested that gut‐microbiome may play critical role in the incidence of CP but relevant clinical evidence are few [12].

We hypothesized that food intake is associated with CP incidence by modulating unique gut‐microbiota, and specific nutrients from food may affect disease progression. To test this hypothesis, we performed gut‐microbiome analysis for CP patient and compared with health subjects or patients of other enterohepatic diseases. And the involvement of specific nutrients was analyzed through dietary pattern questionnaire with patients of single or multiple polyps. Investigation into the triangular connections between “food intake”, “gut‐microbiota”, and “gut‐liver diseases” is now facilitated by advanced taxon set enrichment analysis through analytical platform such as Microbiomeanalyst [13]. Here we studied the association between food intake and gut‐microbiome features in CP patients by comparing gut‐microbiome profiles of patient stool specimen with health subjects. For comparison purpose, we also included patients with other kinds of gut‐liver axis diseases, including alcoholic fatty liver disease (AFLD), metabolic associated fatty liver disease (MFLD), and ulcerative colitis (UC). In addition, to test whether dietary intake of nutrients also affect CP severity we analyzed the daily food choices of CP patients with single or multiple polyps. Our findings add to the growing evidence that dietary intake is associated with the incidence and severity of CP through both impact on gut‐microbiota and nutritional supplies.

## 2 Materials and Methods

### 2.1 Gut‐Microbiome Sequencing

The patient enrollment of this study was approved by the Ethics Committee of the Second Hospital of Shandong University (Ethical Approval Number: KYLL‐2022LW161) and written informed consents were obtained from all the participants. We collected stool specimens from health controls (C, *n* = 15) and patients of alcoholic fatty liver disease (AFLD, *n* = 12), colon polyps (CP, *n* = 29), metabolic associated fatty liver disease (MFLD, *n* = 18) and ulcerative colitis (UC, *n* = 21). Samples were stored in stored in −80°C freezer. Extraction of total fecal genomic DNA that contains 16S rRNA was performed using QIAamp DNA stool mini kits (QIAGEN, Valencia, California). The 16S rRNA gene libraries were prepared using a 2‐step Quadruple‐index PCR method. DNA libraries were constructed through enzymatic DNA fragmentation (200–300 bp), end repair, adapters ligation, and sequence amplification. Briefly, DNA libraries were prepared with qPCR based on Applied Biosystems 7500 Real‐Time PCR System (Thermo Fisher, USA), and the PCR primers were designed based on the sequences of the adapters. The quality of the DNA libraries was assessed using an Agilent 2100 Bioanalyzer (Agilent Technologies, Santa Clara, California). For quality assurance, internal, negative, and positive controls were included in each run. The internal parameters were the specific molecular tag that is placed in the sample before nucleic acid extraction so as to track the entire process and to control the quality of DNA. Negative control sample was introduced with pure sterile water to replace DNA library to monitor any potential contamination. The finally constructed DNA libraries with confirmed quality and i5–i7 dual indexes were pooled and sequenced with Ion PI chip on BioelectronSeq. 4000 (Capitalbio Corporation, Beijing, China) platform. Rawdata in Fastq formats were obtained for further bioinformatics analysis. Sample size was derived from power analysis (https://www.gigacalculator.com/calculators/power-sample-size-calculator.php), with type 1 error rate 5%, power 80%, treatment group 4, standard deviation, and MDE of 1, which reported a number of 10 for each group.

### 2.2 Dietary Pattern Analysis

Dietary pattern questionnaire and analysis have been considered as a promising approach to investigate the association between nutritional intake and disease status. We enrolled 120 CP patients and collected their dietary pattern data. After age and BMI screening, we eventually kept 67 patients, including CP patients with single (*n* = 49) and multiple polyps (*n* = 18). The dietary pattern questionnaire was performed using 6th China Health and Nutrition Survey questionnaire. The transformation from food to nutrients was through China Center for Disease Prevention and Control Database (https://nlc.chinanutri.cn/fq/). The components (per day) include: energy (KJ), protein (g), fat (g), saturated fatty acid (g), mono‐unsaturated fatty acid (g), poly‐unsaturated fatty acid (g), cholesterol (mg), carbohydrate (g), glucose (g), lactose (g), dietary fibre (g), insoluable dietary fiber (g), sodium (mg), vitamin A (μg), vitamin D (μg), vitamin E (mg), vitamin K (mg), vitamin B_1_ (mg), vitamin B_2_ (mg), vitamin B_6_ (mg), vitamin B_12_ (mg), vitamin C (mg), nicotinic acid (mg), folate acid (µg), pantothenic acid (mg), biotin (µg).

The diagnosis of single or multiple CP was processed through enteroscopy from 2021 January to 2022 January. Crown's disease, inflammatory bowel disease, and colon cancer were excluded. The exclusion criteria include “psychological diseases or history”; “impairment of cognitive capacity”, “parents or sibling members carrying colon polyps”, “colorectal cancer”, “schistosomiasis history”, “cancer in first‐degree relatives”, “cholecystectomy history”. Lifestyle (cigarette smoking, alcohol drinking, breakfast), defecation frequency, diagnostic blood biochemistry tests were also collected based on participant willing.

### 2.3 Data Analysis

The original 16 s rRNA sequencing data were first subjected to data quality control step, and the sequences of less than 50 bp and low complexity were removed. Denoising using QIIME 2 [14] was applied to obtain bacterial characteristic representative sequences. Operational Taxanomic Unit (OTU) abundance table is obtained, including the bacterial abundance information of kingdom, phylum, class, order, family, genus, and species. OTU Table was input into Microbiome Analyst on‐line module [13] for Linear discriminant analysis (LDA) effect analysis. LDA (Linear discriminant analysis) effect size analysis (LEFSE) and cladogram were prepared using ImageGP (www.bic.ac.cn/BIC) module with the following parameters: alpha value for the factorial Kruskal‐Wallis test among classes, 0.05; alpha value for the pairwise Wilcoxon test between subclasses = 0.05; threshold on the logarithmic LDA score for discriminative features = 2.0. Metabolic pathway analysis results were also generated from LEFSE. Co‐occurrence analysis was performed using CytoScape [15].

### 2.4 Statistics

All data analysis was performed in SPSS 11.0. Paired T‐test was used for the comparison between two particular groups. Single factor association with CP type (single or multiple) was examined using *χ* ^2^ and for significant data (*p* < 0.05), logistic regression analysis was performed for the factor and relevant factors. Odd ratio and 95% CI (upper and lower boundaries) were calculated from raw data. One‐way ANOVA was used to evaluate the difference among multiple groups, followed by Post‐Hoc Tukey HSD.

## 3 Results

### 3.1 Gut‐Microbiome Features

**Figure 1:**
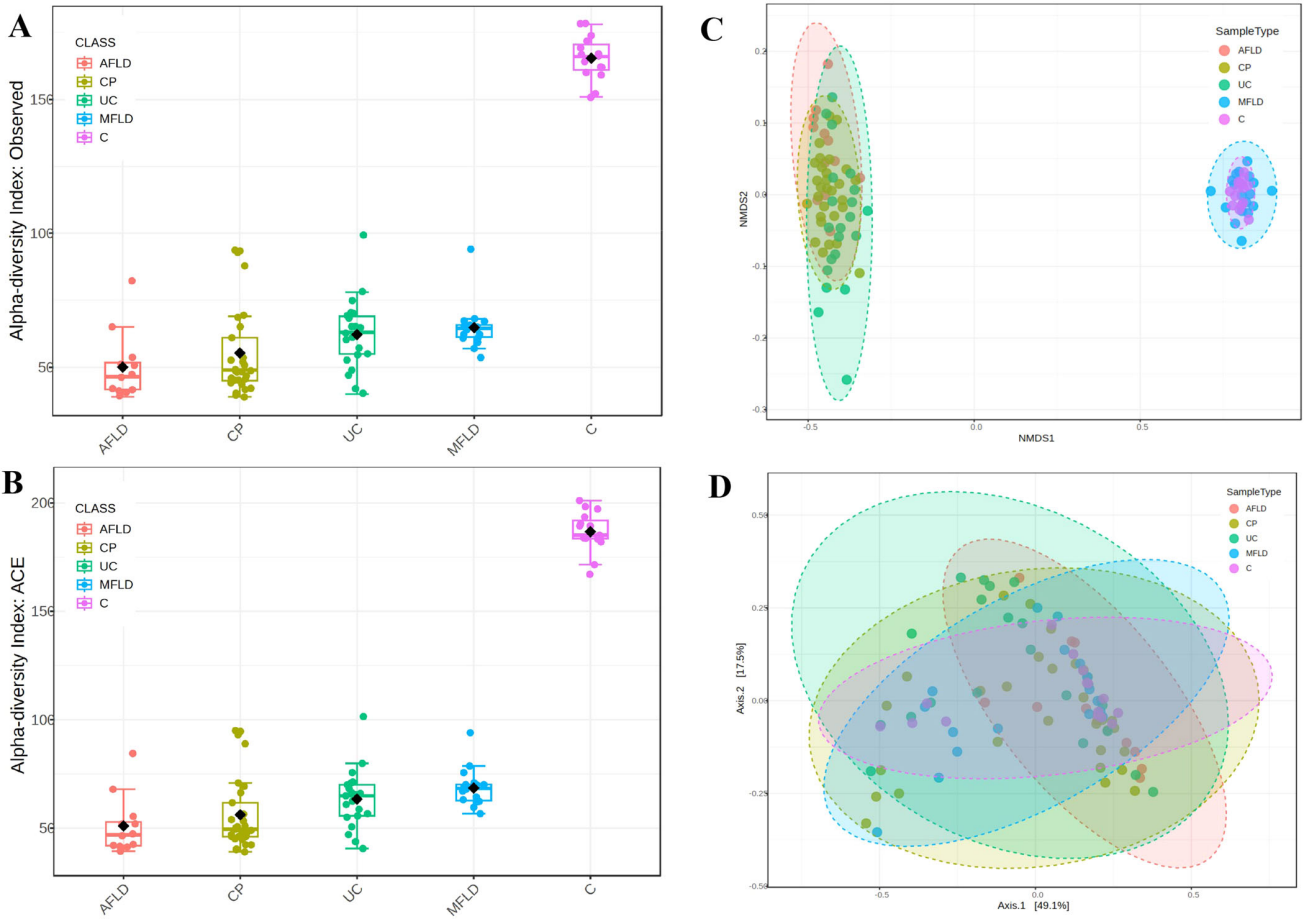
Gut‐microbiome diversity comparison found significant differences between health subjects and the patients with enterohepatic diseases. Alpha diversities and beta diversities of alcoholic fatty liver disease (AFLD), metabolic associated fatty liver disease (MFLD), colon polyps (CP) and ulcerative colitis (UC) and Control (C). The comparisons of alpha diversities were based on observed (Figure 1 A) and ACE (Figure 1 B). The beta diversity was computed with (Figure 1 C) Nonmetric Multidimensional Scaling (NMDS) and (Figure 1 D) Principal Co‐Ordinates Analysis (PcoA) ordination methods.

**Figure 2:**
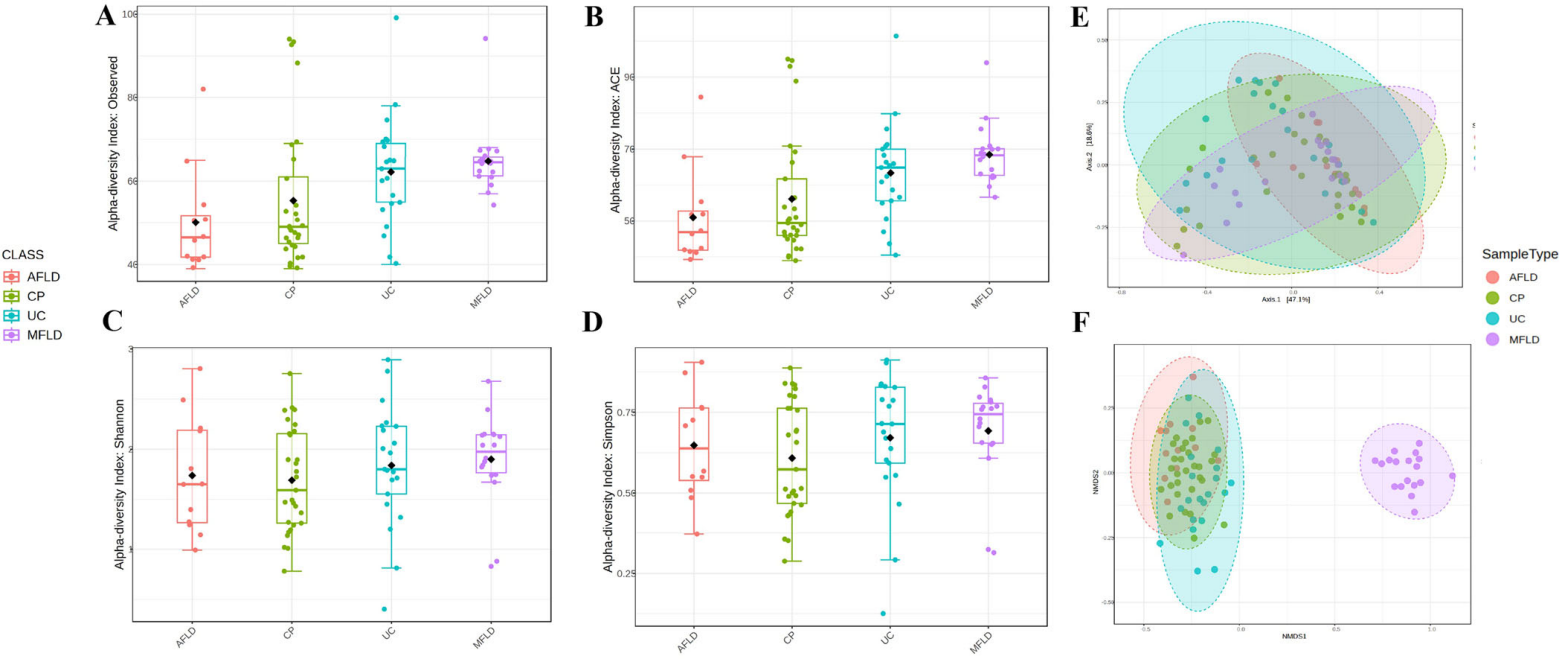
Gut‐microbiome diversities of patients with enterohepatic diseases demonstrated significant differences. Alpha diversities and beta diversities of alcoholic fatty liver disease (AFLD), metabolic associated fatty liver disease (MFLD), colon polyps (CP) and ulcerative colitis (UC). The comparisons of alpha diversities were based on observed (A), ACE (B), Simpson (C) and Fisher (D) indexes. The beta diversity was computed with (E) Nonmetric Multidimensional Scaling (NMDS) and (F) Principal Co‐Ordinates Analysis (PcoA) ordination methods.

The gut‐microbiome structures of patients with enterohepatic diseases are very different with healthy subjects. The gut‐microbiome of CP, AFLD, MFLD, and UC are highly different compared with healthy subjects (C) (Figure 1 A–B). We compared the alpha diversities of the 4 groups and we found significantly higher alpha diversity in C group (*p* < 0.001). The alpha‐diversity of MFLD (ACE 67.89) was higher than AFLD (ACE 50.69, *p* < 0.001) and CP (ACE 56.02, *p* < 0.001), but no statistical significance was found in UC (ACE 63.24, *p* = 0.17). The beta‐diversity showed again the separation between C with the disease groups (Figure 1 C–D). We first compared the alpha diversities of the four groups using four indexes, naturally observed, ACE, Simpson, and Shannon (Figure 2 A–D). Of the four indexes, naturally observed and ACE metrics demonstrated are the largest differences of CP, AFLD, UC, and MFLD groups, and there was significant difference for one‐way ANOVA (F = 5.1417, *p* = 0.003). Major difference of naturally observed and ACE metrics exists between AFLD and MFLD groups (*p* < 0.001). The alpha diversity of the gut‐microbiome of CP patients did not show any remarkable non‐similarity with AFLD and UC groups. The other metrics such as Shannon, Simpson or Fisher, did not show any difference with statistical significance. The beta diversity (Figure 2 E–F) was computed and ranked by Bray‐Cutis distance with both Principal Co‐Ordinates Analysis (PcoA) and Nonmetric Multidimensional Scaling (NMDS) ordination methods. The between‐habitat diversity (beta‐diversity) with NMDS method showed PERMANOVA *F*‐value 3.0539; R‐squared 0.10758; *p*‐value 0.003; [NMDS] Stress = 0.12448 and PCoA showed PERMANOVA *F*‐value 3.0539; R‐squared 0.10758; *p*‐value 0.003.

**Figure 3:**
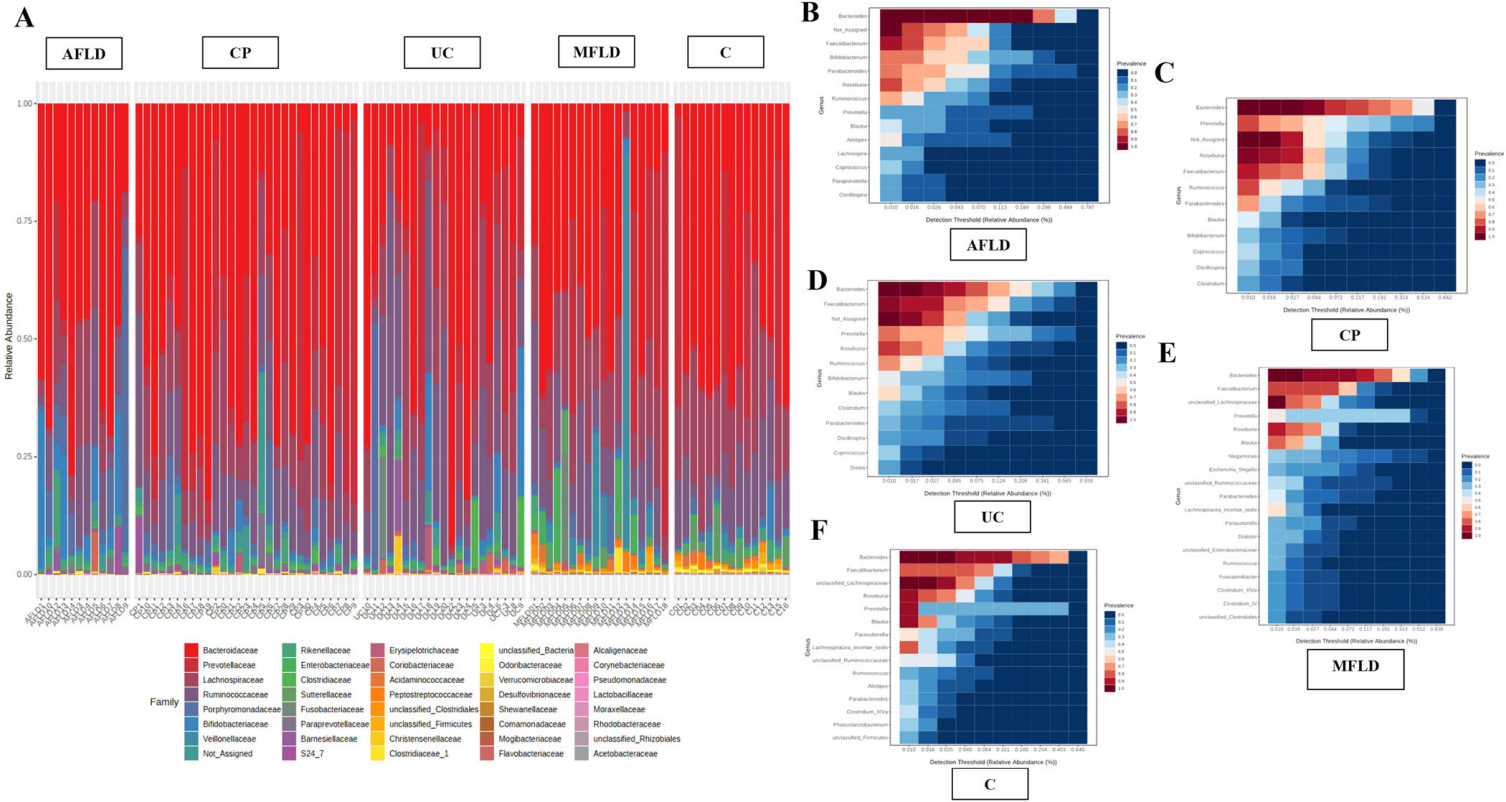
Patients with four enterohepatic diseases have unique core gut‐microbiome and unsimilar abundances of gut‐microbiome profiles. This may be associated their etiology. The family‐level microbiome taxa with relative abundance. (A) Accumulative histogram for family‐level microbiome taxa of alcoholic fatty liver disease (AFLD), metabolic associated fatty liver disease (MFLD), colon polyps (CP) and ulcerative colitis (UC). (B) Dominant gut‐microbiome families shown in alcoholic fatty liver disease patients. (C) Dominant gut‐microbiome families shown in colon polyps. (D) Dominant gut‐microbiome families shown in ulcerative colitis. (E) Dominant gut‐microbiome families shown in metabolic associated fatty liver disease. (F) Heath control core microbiome.

The accumulative histogram for taxa abundances is shown in Figure 3. Interestingly, we found that the phylum richness differed largely in the AFLD, MFLD CP, and UC groups. The gut‐microbiota of alcoholic fatty liver disease patients were occupied by Actinobacteria (AFLD, 3487.92; MFLD, 264.5; CP, 489.45; UC, 634.38, Figure 3). One‐way ANOVA showed that there was significant difference of Actinobacteria richness existing among AFLD, UC and CP (*F* = 5.39, *p* = 0.0006). Post‐hoc Tukey HSD showed that Actinobacteria had a dominant abundance in the gut‐microbiota of AFLD patients compared with colon polyps (*p* < 0.001). The abundances for different phylum are shown in Table 1, which presents the t‐test that we performed between each pair of AFLD, MFLD, CP, UC, and C groups. Besides, we also performed *F*‐test for the differential phylum among disease groups. Bacteroidetes demonstrated significant preference in different disease conditions (ANOVA, *F* = 12.70, *p* < 0.001). Its dominance in colon polyps was much higher than the other two diseases (*p* < 0.05 between AFLD and CP, but not significant between CP and U). Alcoholic fatty liver disease patients have significantly lower abundance of *Bacteroidetes* than the other three diseases (*p* < 0.05). By contrast, the gut‐microbiota of CP and MFLD patients showed higher *Bacteroidetes* than AFLD and UC. The dominant phylum of gut‐microbiota in ulcerative colitis and metabolic associated fatty liver disease patients were *Firmicutes*. The *Firmicutes* in ulcerative colitis patients had significant difference only between alcoholic fatty liver disease and ulcerative colitis. However, there were significant differences in the intestinal flora of *Firmicutes* between MFLD patients and the other three disease patients. In general, the phylum composition at global level in the four disease conditions showed major divergences in the four enterohepatic diseases.

**Table 1:** Phylum abundances identified from patients enrolled for gut‐microbiome analysis.

Group	Statistics	Actinobacteria	Bacteroidetes	Firmicutes	Fusobacteria	Proteobacteria	Verrucomicrobia
Control	average	706.80	41330.07	13509.07	20.13	0.00	92.33
	SD	1751.60	4087.16	5259.26	37.76	0.00	231.60
Alcoholic fatty liver disease	average	3487.92	21571.33	7492.33	90.67	119.17	4.17
	SD	4163.73	7221.72	5430.93	167.68	146.53	11.38
	*p* value vs. C	0.03	< 0.0001	0.01	0.14	0.01	0.22
Colon polyps	average	489.45	26721.72	10695.48	34.14	686.97	22.86
	SD	843.38	9054.13	6474.57	98.55	2521.90	94.67
	*p* value vs. C	0.59	< 0.0001	0.16	0.60	0.30	0.18
	*p* value vs. AFLD	< 0.0001	0.09	0.15	0.20	0.44	0.50
Ulcerative colitis	average	1634.38	20850.00	16391.62	590.00	68.05	5.14
	SD	2595.96	12550.68	11195.48	2203.41	51.39	8.48
	*p* value vs. C	0.25	< 0.0001	0.37	0.34	< 0.0001	0.10
	*p* value vs. AFLD	0.14	0.86	0.02	0.45	0.17	0.79
	*p* value vs. CP	0.03	0.06	0.03	0.19	0.27	0.40
Metabolic associated fatty liver disease	average	264.50	33296.72	23266.83	852.39	3235.50	46.44
	SD	382.18	11676.99	8969.01	3561.52	3794.25	140.85
	*p* value vs. C	0.31	0.01	0.66	0.37	0.41	0.50
	*p* value vs AFLD	0.004	0.005	< 0.0001	0.47	0.009	0.31
	*p* value vs. CP	0.29	0.03	< 0.0001	0.22	0.008	0.49
	*p* value vs. UC	0.04	0.003	0.04	0.78	0.0005	0.19

**Figure 4:**
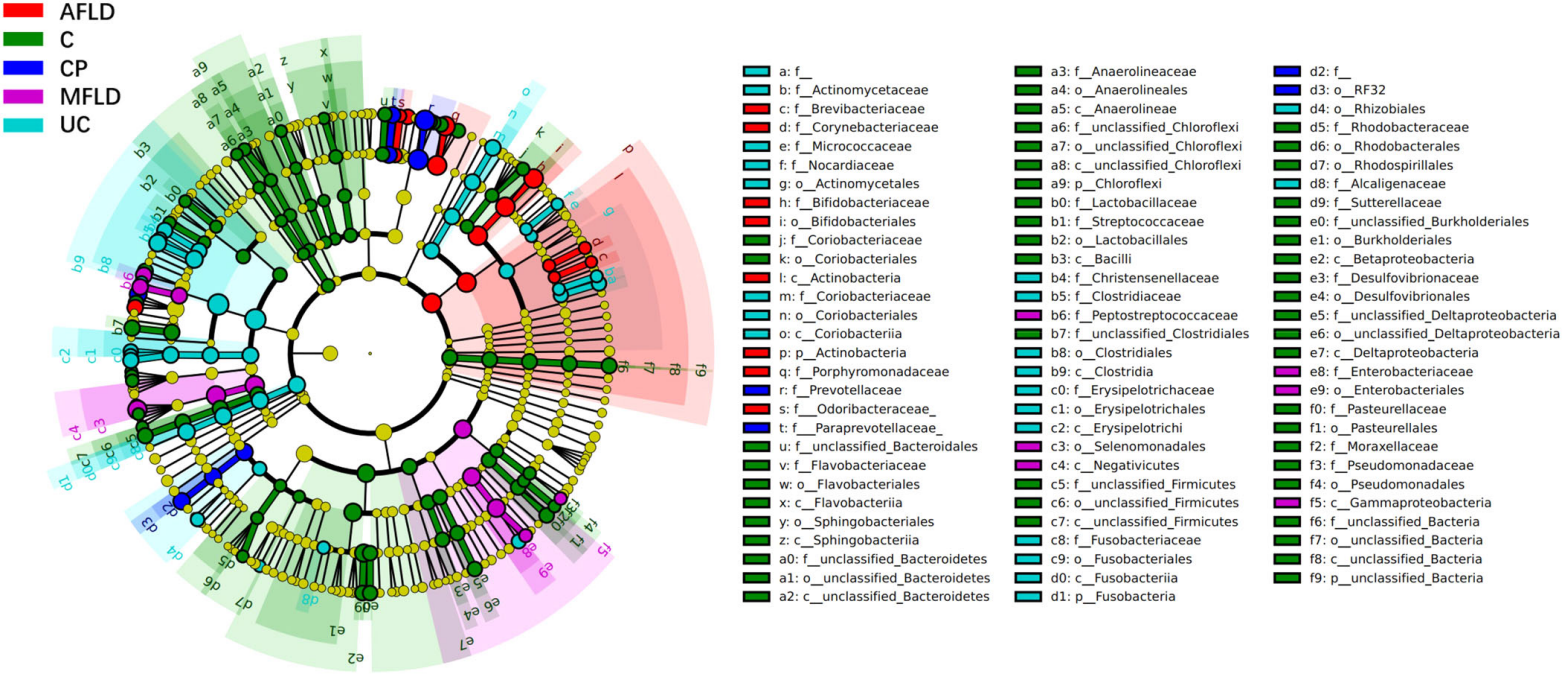
Illustration of unique gut‐microbiome in patients of four enterohepatic diseases. LEfSe (Linear discriminant analysis Effect Size) analysis was performed to identify disease‐specific gut microbiome signatures among patients with alcoholic fatty liver disease (AFLD), metabolic associated fatty liver disease (MAFLD), colon polyps (CP), ulcerative colitis (UC), and healthy controls. The analysis revealed distinct microbiome families enriched in each disease group.

The most abundant microbial families cross the disease groups were *Bacteroidaceae*, *Ruminococcaceae*, *Prevotellaceae,* and *Lachnospiraceae*. The core microbial genera in the AFLD were *Bacteroides*, *Faecalibacterium*, *Bifidobacterium*, *Parabacteroides*, *Roseburia* (Figure 3 B); in CP were *Bacteroides*, *Prevotella*, *Roseburia*, *Faecalibacterium*, *Ruminococcus*, *Parabacteroides* (Figure 3 C); in UC were *Bacteroides*, *Faecalibacterium*, *Prevotella*, *Roseburia*, *Ruminococcus*, *Bifidobacterium*, *Blautia* (Figure 3 D); in MFLD were *Bacteroides*, *Faecalibacterium*, *Prevotella*, *Roseburia*, and *Blautia* (Figure 3 E). The major differential gut‐microbiome taxon is organized in Table 2. The remarkable gut‐microbiome metabolic pathways were identified between UC and CP groups. The functional analysis of gut‐microbiome showed that the gut‐microbiome in CP patients may potentially elevate the chances of cardiac circulation and Colorectal cancer, compared with the UC group (Table 3). And for UC gut‐microbiome it seems the gut‐microbiome may not affect patients' health directly, yet exerting impact on the metabolism and signaling pathways. Further investigation into the “unique” microbiome using LEFSE (Linear discriminant analysis Effect Size) method (Figure 4) showed that C and UC maintained more unique species of gut‐microbiome than AFLD, MFLD, and CP. For the AFLD and CP groups, the most differential microbial family higher in AFLD but lower in CP is *Bifidobacteriaceae*, followed by *Porphyromonadaceae* and *Odoribacteraceae*. Importantly, *Prevotellaceae*, *Paraprevotellaceae* showed higher richness in CP group only.

**Table 2:** Major gut‐microbiome family abundance compared between healthy subjects and patients with enterohepatic diseases.

Gut‐microbiome family	Control	Alcoholic fatty liver disease			Colon polyps			Ulcerative colitis			Metabolic associated fatty liver disease		
	Average	Average	Fold	*p* value	Average	Fold	*p* value	Average	Fold	*p* value	Average	Fold	*p* value
Alcaligenaceae	0.002 ± 0.002	0.009 ± 0.011	5.5	0.032	0.009 ± 0.011	5.5	0.032	0.04 ± 0.075	26.02	0.05	< 0.001 < 0.001	0	< 0.001
Bacteroidaceae	42.163 ± 19.788	48.764 ± 19.688	1.16	0.414	48.764 ± 19.688	1.16	0.414	44.634 ± 26.272	1.06	0.756	34.950 ± 18.296	0.83	0.216
Barnesiellaceae	14.682 ± 8.997	0.511 ± 0.969	0.03	< 0.001	0.511 ± 0.969	0.03	< 0.001	0.243 ± 0.32	0.02	< 0.001	< 0.001 < 0.001	0	< 0.001
Bifidobacteriaceae	0.995 ± 2.546	8.979 ± 10.367	9.03	0.01	8.979 ± 10.367	9.03	0.01	1.145 ± 1.765	1.15	0.825	0.470 ± 0.776	0.47	0.413
Christensenellaceae	0.459 ± 0.626	0.005 ± 0.015	0.01	0.023	0.005 ± 0.015	0.01	0.023	0.051 ± 0.111	0.11	0.002	< 0.001 < 0.001	0	< 0.001
Clostridiaceae	0.122 ± 0.087	0.202 ± 0.124	1.66	0.07	0.202 ± 0.124	1.66	0.07	0.622 ± 0.655	5.1	0.006	< 0.001 < 0.001	0	< 0.001
Coriobacteriaceae	0.085 ± 0.093	0.723 ± 2.257	8.49	0.303	0.723 ± 2.257	8.49	0.303	0.067 ± 0.086	0.79	0.539	< 0.001 < 0.001	0	< 0.001
Corynebacteriaceae	0.013 ± 0.004	0.035 ± 0.061	2.71	0.188	0.035 ± 0.061	2.71	0.188	0.022 ± 0.047	1.67	0.489	< 0.001 < 0.001	0	0.000
Enterobacteriaceae	0.499 ± 0.311	0.154 ± 0.196	0.31	0.003	0.154 ± 0.196	0.31	0.003	0.046 ± 0.076	0.09	< 0.001	0.083 ± 0.100	0.17	0.457
Erysipelotrichaceae	0.318 ± 0.242	0.061 ± 0.058	0.19	0.002	0.061 ± 0.058	0.19	0.002	0.202 ± 0.664	0.64	0.526	3.276 ± 5.401	10.30	0.058
Fusobacteriaceae	0.029 ± 0.055	0.295 ± 0.548	10.14	0.084	0.295 ± 0.548	10.14	0.084	0.105 ± 0.284	3.6	0.323	0.287 ± 0.431	9.90	0.289
Lachnospiraceae	18.339 ± 7.462	10.969 ± 9.306	0.6	0.037	10.969 ± 9.306	0.6	0.037	13.218 ± 6.937	0.72	0.033	1.289 ± 5.381	0.07	0.373
Lactobacillaceae	0.034 ± 0.033	< 0.001 < 0.001	0	0.002	< 0.001 < 0.001	0	0.002	0.053 ± 0.061	1.57	0.274	16.312 ± 6.812	479.76	0.369
Mogibacteriaceae	0.611 ± 0.518	0.045 ± 0.089	0.07	0.001	0.045 ± 0.089	0.07	0.001	0.126 ± 0.202	0.21	< 0.001	< 0.001 < 0.001	0	0.000
Odoribacteraceae	0.026 ± 0.012	0.126 ± 0.159	4.8	0.028	0.126 ± 0.159	4.8	0.028	1.055 ± 2.076	40.01	0.068	< 0.001 < 0.001	0	< 0.001
Paraprevotellaceae	0.448 ± 0.110	0.7 ± 0.762	1.56	0.236	0.7 ± 0.762	1.56	0.236	0.054 ± 0.125	0.12	< 0.001	< 0.001 < 0.001	0	< 0.001
Peptostreptococcaceae	0.302 ± 0.735	0.042 ± 0.064	0.14	0.251	0.042 ± 0.064	0.14	0.251	2.797 ± 4.55	9.25	0.046	< 0.001 < 0.001	0	< 0.001
Porphyromonadaceae	1.657 ± 0.884	10.217 ± 16.745	6.17	0.069	10.217 ± 16.745	6.17	0.069	19.007 ± 25.47	11.47	0.014	0.771 ± 1.176	0.47	0.212
Prevotellaceae	14.234 ± 21.132	2.477 ± 5.61	0.17	0.083	2.477 ± 5.61	0.17	0.083	1.034 ± 1.21	0.07	0.002	1.955 ± 2.811	0.14	0.428
Rikenellaceae	0.860 ± 1.064	2.17 ± 3.699	2.52	0.221	2.17 ± 3.699	2.52	0.221	12.221 ± 7.484	14.2	< 0.001	16.608 ± 24.757	19.31	0.778
Ruminococcaceae	12.306 ± 6.027	11.208 ± 7.804	0.91	0.695	11.208 ± 7.804	0.91	0.695	0.554 ± 2.103	0.05	< 0.001	0.370 ± 0.887	0.03	0.149
S24_7	0.024 ± 0.013	0.932 ± 2.67	38.66	0.217	0.932 ± 2.67	38.66	0.217	0.03 ± 0.076	1.22	0.792	11.785 ± 9.143	491.04	0.727

*Note: p*‐value suggests significance between the control subjects and cases.

**Table 3:** Functional analysis for the major difference between CP and UC.

Elevated in colon polyps		Elevated in ulcerative colitis	
Pathway and disease	CP/UC	Pathway and disease	UC/CP
Cardiac muscle contraction	18.35	Cell cycle	38.42
Colorectal cancer	7.90	Measles	38.42
p53 signaling pathway	7.90	mTOR signaling pathway	38.42
Small cell lung cancer	7.90	mRNA surveillance pathway	30.48
Viral myocarditis	7.90	Biosynthesis of type II polyketide backbone	28.24
Toxoplasmosis	7.89	Betalain biosynthesis	23.53
Influenza A	6.73	Indole alkaloid biosynthesis	22.48
Photosynthesis ‐ antenna proteins	6.29	Hypertrophic cardiomyopathy (HCM)	21.23
Apoptosis	5.98	Renin‐angiotensin system	17.10
Calcium signaling pathway	5.79	Vibrio cholerae infection	16.80
Fatty acid elongation in mitochondria	3.92	Phagosome	15.88
Systemic lupus erythematosus	3.19	Stilbenoid, diarylheptanoid and gingerol biosynthesis	12.06
Bacterial invasion of epithelial cells	2.39	Endocytosis	11.05
Renal cell carcinoma	2.23	Fc gamma R‐mediated phagocytosis	11.05
Isoflavonoid biosynthesis	1.81	GnRH signaling pathway	11.05
Huntington's disease	1.72	Bile secretion	10.49
Glycosphingolipid biosynthesis	1.72	Bladder cancer	8.73
Lipopolysaccharide biosynthesis	1.32	G protein‐coupled receptors	8.29

### 3.2 Food Intake Association

**Figure 5:**
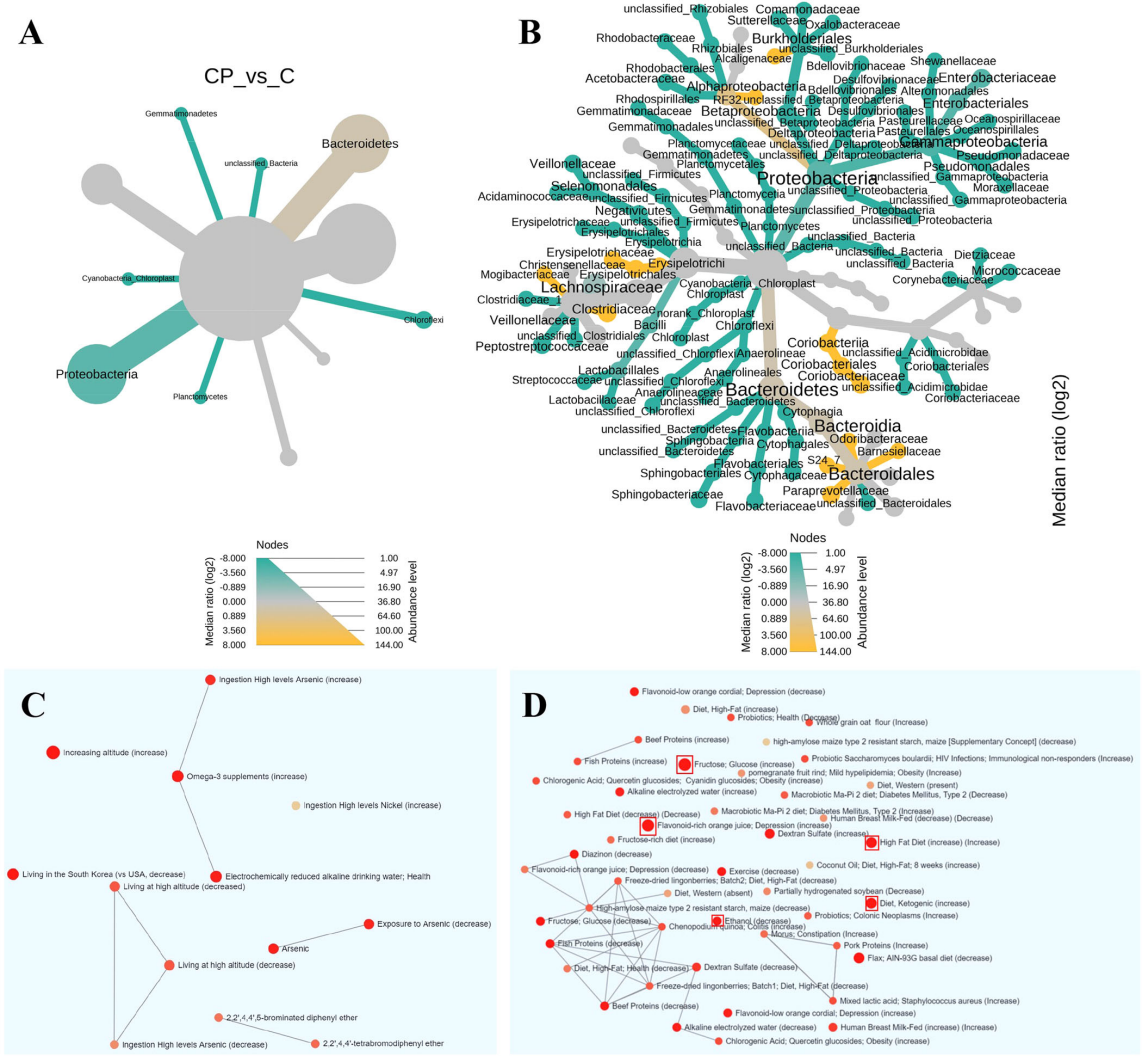
Global comparison between CP patients and healthy subjects, and the food intake association indicated by the comparison between the patients and healthy controls. (A) heat‐tree analysis in the healthy control group the colon polyps group. (A) phylum heat‐tree. (B) families heat‐tree. (C‐D) Taxon Set Enrichment Analysis based on Gut‐microbiome Family.

To understand the potential impact of food intake on gut‐microbiome features. We first input the health controls and CP‐enriched gut‐microbiota (Table 4) into the comparative heat tree analysis using MicrobiomeAnalyst (https://www.microbiomeanalyst.ca/). Shown in Figure 5 A–B, the CP patients have more Bacteroidetes (abundance level), such as Odoribacteraceae, S24_7, Barnesiellaceae, and Paraprevotellaceae. These gut‐microbiome have shown metabolic functions on high‐calories and high‐fat foods. Their elevation in CP patients may be caused by unhealthy dietary pattern. Next, we performed taxon association analysis with environmental exposure, dietary intake, and medication. Shown in Figure 5 C–D, by taxon association analysis we found that CP patient gut‐microbiome was associated with dietary exposure to environmental contaminants (arsenic and PBDE, polybrominated diphenyl ethers), high‐fat and ketogenic diets, fructose intake, and glucose intake. Protein intake showed complex associations, probably caused by variations from different laboratory experiments. Notably, the taxon association can only indicate the “association” but not the causation. The association contains both “increase” and “decrease” links, because gut‐microbial species within same family may have diverse response to same factor. Causation for dietary intake, gut‐microbiome, and the disease incidence should be investigated through epidemiologic cohort studies.

**Table 4:** Basic information of colon polyps patients.

Information	Single polyps	Multiple polyps
BMI (mean, SD)	27.39 ± 16.1	24.76 ± 2.5
Age (mean, SD)	55.56 ± 10.9	58.43 ± 8.22
Male %	67.82%	68.57%
Female %	32.18%	31.43%
Marriage %	88.37%	100%

**Figure 6:**
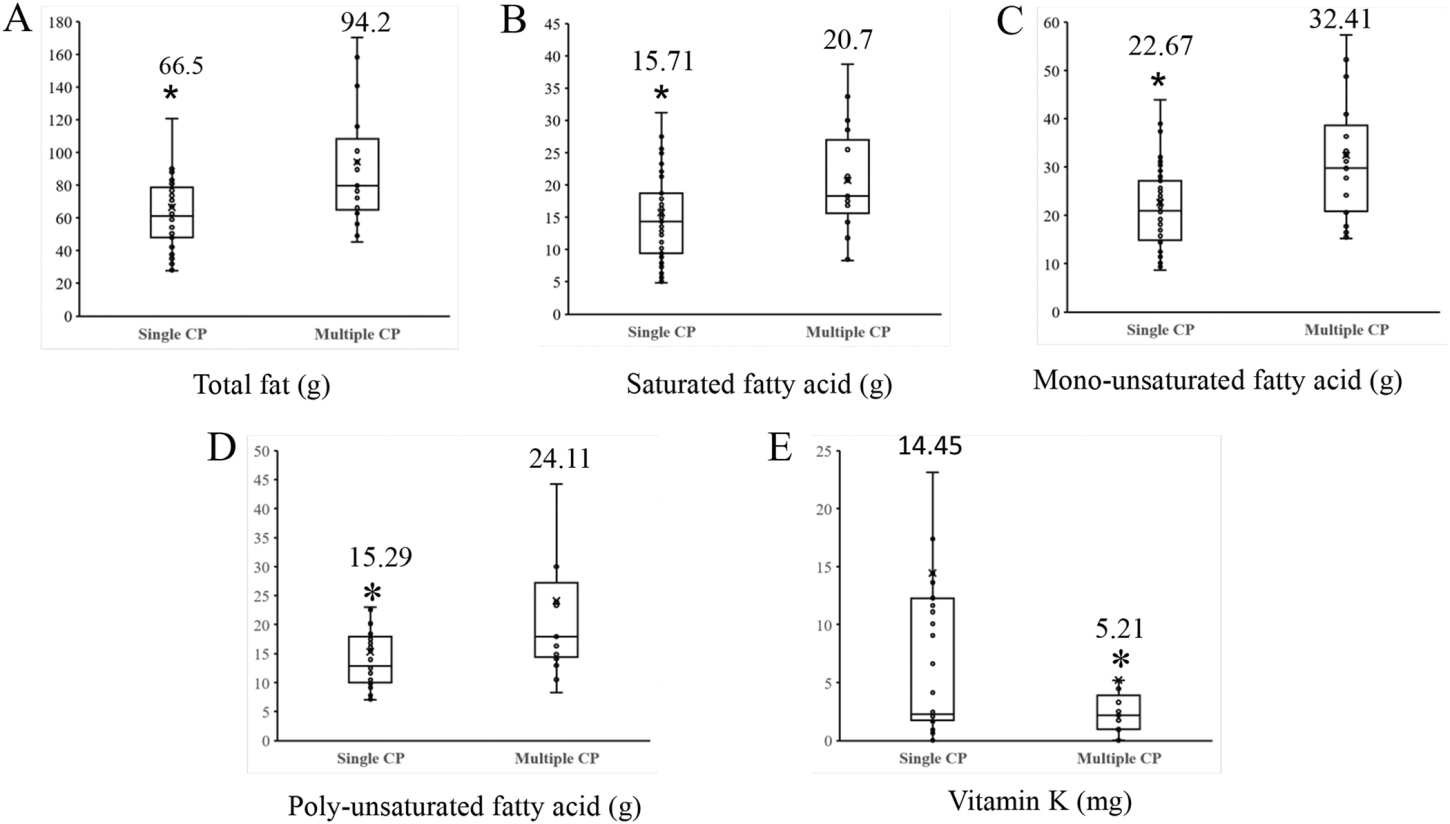
Dietary patten analysis shows the significant differences of food intake fat, saturated and unsaturated fatty acids, and consumption of Vitamin E. Differential nutrients between dietary patterns of patients of multiple CP (*n* = 59) and single CP (*n* = 18) are shown in (A) total fat intake, (B) saturated fatty acid, (C) mono‐unsaturated fatty acid, (D) poly‐unsaturated fatty acid, (E) Vitamin E.

To test whether dietary pattern also affects disease severity, we choose CP patients with single or multiple polyps as study targets to compare. The patient dietary patterns were investigated through food questionnaire. We found that multiple CP patients demonstrated significantly higher intake of fat but less vitamin E/K. The results are shown in Figure 6. Saturated fatty acid, mono‐/poly‐unsaturated fatty acid all showed higher consumption level in the multiple CP group.

## 4 Discussion

In this study our gut‐microbiome analysis and dietary pattern analysis demonstrated that food intake is associated with CP incidence by forming featured gut‐microbiota, and specific nutrients from food is associated with CP severity. Though the sample size is not large but our analysis add to the current growing evidence that food intake affect CP incidence and progression.

### 4.1 Disease‐Featured Gut‐Microbiome

The term gut‐liver axis was formally stated by Marshall in his publication on 1998, which emphasized on the connection between liver and gut, and later also incorporated gut‐microbiota [16]. The major diseases related with gut‐liver axis include colon polyps (CP), alcoholic fatty liver disease (AFLD), metabolic associated fatty liver disease (MAFLD) and ulcerative colitis (UC) [17, 18]. It is well recognized that the incidence of gut‐liver axis diseases is closely associated with dietary intake [19, 20]. However, relevant data are far less than enough to support the emerging findings and hypothesis from laboratory experiments [21].

Gut‐microbiome diversities differ largely (Figure 1), and AFLD showed worst diversity. It is well‐known that in alcohol drinker the diversity and function of gut‐microbiota are both largely impared [22]. The alpha‐diversity between UC and CP were very close, yet difference of their beta‐diversities suggest that the gut‐microbiome of UC and CP were formed in different disease and physiological environments (Figure 2 E F) [23].

Indeed, the disease status formulated different gut‐microbiome colonies. We found the disease statuses all have unique dominant gut‐microbial phylums. AFLD is featured by Actinobacteria, whereas the gut‐microbiome in CP patients is dominated by Bacteroidetes, the gut‐microbiome in MFLD patients is dominated by Proteobacteria and the gut‐microbiome in UC patients is dominated by Firmicutes (Figure 3). Actinobacteria is known as the indicator of dysbiosis, and in AFLD the dominance of Actinobacteria may be caused by alcohol toxicity [24].

Further analysis of relative abundance at family level revealed that Bacteroidaceae, Ruminococcaceae, Lachnospiraceae, and Prevotellaceae exist in all four types of enterohepatic diseases at relative high abundance (Figure 3 A). The first three gut‐microbial families all provide important immune/metabolic functions to human host, such as synthesis of short chain fatty acids [25, 26]. But Prevotellaceae is associated with induction of inflammation in digestive tract [27]. For the core gut‐microbiome (Figure 3 B C D E), we found that generally the dominant beneficial gut‐microbial families still exist in the patients, which were in line with the observation on the accumulative histogram (Figure 3 A). However, Prevotella and Paraprevotella remarked the gut‐microbiome of CP patients, which suggest severe inflammation that may be induced by colon polyps.

### 4.2 CP and UC‐Enriched Gut‐Microbiome: Similarity and Difference

The gut‐microbiome structure of CP and UC seemed to be close yet by using LEFSE analysis we found that the gut‐microbiome of the two disease conditions were totally different. The functional analysis of gut‐microbiome showed that the gut‐microbiome in CP patients may potentially elevate the chances of cardiac circulation and Colorectal cancer, compared with the UC group (Table 3). And for UC gut‐microbiome it seems the gut‐microbiome may not affect patients' health directly, yet exerting impact on the metabolism and signaling pathways.

We next turn to LEFSE method to evaluate the uniqueness of microbiome families in different diseases and control group (Figure 4). The healthy subjects have highest abuncance and diversities in Bacteroidaceae. Following health subjects (C), UC hosts most “unique” families, which is in consistent with alpha‐diversity observation (Figure 2). We found high abundances of Prevotella and Paraprevotella in the gut‐microbiome of CP patients (Figure 4). The LEFSE scores of Prevotella and Paraprevotella were both high among four disease conditions. Interestingly, we observed that Porphyromonadaceae, a family belong to Bacteroidetes order, dominates the gut‐microbiome of AFLD patients. This microbial family is associated with infection in oral cavity and digestive tract in human [28]. The functional metabolic analysis in Table 3 also showed that there were remarkable un‐similarities between CP and U patients, even both of them were gut‐inflammatory diseases. The colorectal cancer risk is highly enriched in CP patients compared with UC patients. Our study is consistent with available publications on seven families and we also provide novel findings for the other families that are linked with CP.

### 4.3 Food Intake Impact on CP Incidence and Severity

Finally, as a summary we presented the heat‐tree analysis in Figure 5. Bacteroidetes demonstrated significant high level in the control group and Proteobacteria showed to be the most abundant phylum in the CP group. More specific families can be seen in Figure 5. We also performed Taxon Set Enrichment Analysis based on Gut‐microbiome Family. Environmental, medication, and dietary levels of intestinal flora were analyzed separately. Specifically, exposure to Arsenic and PBDE was significantly correlated with *Porphyromonadaceae*, *Christensenellaceae*, *Lachnospiraceae*, *Ruminococcaceae,* and *Erysipelotrichaceae* (*p* < 0.01). Diet, Ketogenic (increase) had significant correlation with *Bacteroidaceae*, *Erysipelotrichaceae*, *Lachnospiraceae,* and *Peptostreptococcaceae* (*p* < 0.001). High Fat Diet (increase) had significant correlation with *Coriobacteriaceae*, *Erysipelotrichaceae*, *Mogibacteriaceae*, *Ruminococcaceae* (*p* < 0.01). Beyond this, seen from Figure 6, the dietary patten analysis found that the consumption of total fat, saturated fatty acid and unsaturated fatty acid in multiple CP group were all significantly higher than single CP. This matches with the epidemiologic findings reported by Mari et al [29]. Lower intake of vitamin E was also reported before, as one of risk factor shared by CP and obesity [30]. Specific dietary pattern data is provided in Supporting Information.

## 5 Conclusion

To summarize, gut‐microbiome in the patients with colon polyps demonstrated to have remarkable disease‐specific and food‐intake associated composition. The CP gut‐microbiome features are unique not only by comparing with healthy subjects, but also when compared with alcoholic fatty liver disease (AFLD), metabolic associated fatty liver disease (MFLD), CP, and ulcerative colitis (UC). The CP patients demonstrated gut‐microbiome that may be affected by high‐fat diet, high‐caloric diet, ketogenic diet, and too much consumption of fructose. *Prevotellaceae*, *Paraprevotellaceae* showed higher richness in CP group only. This suggests their potential as microbial biomarkers for CP diagnosis or severity stratification, warranting further investigation into their functional and metabolic roles. Dietary pattern analysis further showed that food intake may also be involved with CP severity, as patients of multiple CP consume more fat, more saturated/unsaturated fatty acids, but less vitamin E than the patients with single CP. Our findings are consistent with related nutritional studies and this study provide key gut‐microbiome taxonomies that might be the targets of unhealthy diets in CP incidence and development.

## Author Contributions

**Chuanmin Ma:** formal analysis, writing – original draft, software, data curation. **Mingbao Zhang:** methodology, writing – original draft. **Binbin Chen:** data curation. **Ming Liu:** methodology. **Huiyu Jiang:** validation, methodology. **Jiaorong Li:** formal analysis. **Xintong Song:** supervision. **Xiangrui Wei:** funding acquisition, visualization. **Zhixing Wei:** project administration, investigation. **Jingyao Liu:** resources. **Hengyun Guan:** conceptualization. **Jun Zhou:** writing – review and editing, conceptualization. **Hui Liu:** writing – review and editing, conceptualization.

## Ethics Statement

This study was approved by the Ethics Committee of the Second Hospital of Shandong University (Ethical Approval Number: KYLL‐2022LW161).

## Consent

Written informed consents were obtained from all the participants.

## Conflicts of Interest

The authors declare no conflicts of interest.

## 1 Transparency Statement

The lead author Hui Liu affirms that this manuscript is an honest, accurate, and transparent account of the study being reported; that no important aspects of the study have been omitted; and that any discrepancies from the study as planned (and, if relevant, registered) have been explained.

## Supporting information


**SI 1:** Basic Information Lifestyle.
**SI 2:** Basic Information tests.
**SI 3:** LEFSe. **SI 4**.

## Data Availability

The data that support the findings of this study are available from the corresponding author upon reasonable request.
